# Social Media for e-Government in the Public Health Sector: Protocol for a Systematic Review

**DOI:** 10.2196/resprot.5421

**Published:** 2016-03-11

**Authors:** Massimo Franco, Aizhan Tursunbayeva, Claudia Pagliari

**Affiliations:** ^1^ Department of Economics, Management, Society, and Institutions University of Molise Campobasso Italy; ^2^ eHealth Research Group Usher Institute of Population Health Sciences and Informatics University of Edinburgh Edinburgh United Kingdom

**Keywords:** e-government, eHealth, health organizations, social media

## Abstract

**Background:**

Public sector organizations worldwide are engaging with social media as part of a growing e-government agenda. These include government departments of health, public health agencies, and state-funded health care and research organizations. Although examples of social media in health have been described in the literature, little is known about their overall scope or how they are achieving the objectives of e-government. A systematic literature review is underway to capture and synthesize existing evidence on the adoption, use, and impacts of social media in the public health sector. A series of parallel scoping exercises has taken place to examine (1) relevant existing systematic reviews, to assess their focus, breadth, and fit with our review topic, (2) existing concepts related to e-government, public health, and the public health sector, to assess how semantic complexity might influence the review process, and (3) the results of pilot searches, to examine the fit of social media within the e-government and health literatures. The methods and observations of the scoping exercises are reported in this protocol, alongside the methods and interim results for the systematic review itself.

**Objective:**

The systematic review has three main objectives: To capture the corpus of published studies on the uses of social media by public health organizations; to classify the objectives for which social media have been deployed in these contexts and the methods used; and to analyze and synthesize evidence of the uptake, use, and impacts of social media on various outcomes.

**Methods:**

A set of scoping exercises were undertaken, to inform the search strategy and analytic framework. Searches have been carried out in MEDLINE, the Cochrane Library, Web of Science, and the Scopus international electronic databases, and appropriate gray literature sources. Articles published between January 1, 2004, and July 12, 2015, were included. There was no restriction by language. One reviewer (AT) has independently screened citations generated by the search terms and is extracting data from the selected articles. A second author (CP) is cross-checking the outputs to ensure the fit of selected articles with the inclusion criteria and appropriate data extraction. A PRISMA flow diagram will be created, to track the study selection process and ensure transparency and replicability of the review.

**Results:**

Scoping work revealed that the literature on social media for e-government in the public health sector is complicated by heterogeneous terminologies and concepts, although studies at the intersection of these three topics exist. Not all types of e-government are evident in the health care literature. Interim results suggest that most relevant articles focus on usage alone.

**Conclusions:**

Public health organizations may be taking it for granted that social media deliver benefits, rather than attempting to evaluate their adoption or impacts. Published taxonomies of e-government hold promise for organizing and interpreting the review results. The systematic review is underway and completion is expected in the beginning of 2016.

**Trial Registration:**

PROSPERO International Prospective Register of Systematic Reviews: CRD42015024731; http://www.crd.york.ac.uk/PROSPERO/display_record.asp?ID=CRD42015024731 (Archived by WebCite at http://www.webcitation.org/6dV1Cin91).

## Introduction

### e-Government, Social Media, and the Health Sector

Governments around the world are being challenged by increasing public demand for institutional transparency, involvement in decision making, and easier access to services, as well as by the financial imperative for greater efficiencies in the business of government itself [1]. To respond to these challenges, they have sought to harness the Internet and related information and communications technologies for sharing information and enabling transactions between governmental bodies, businesses, and citizens, as part of a broader “e-government” agenda [1,2]. One channel through which this is being approached is social media, including networking and dissemination platforms, such as Facebook and Twitter, and bespoke online tools for eliciting feedback and other e-government objectives [3,4]. According to a recent United Nations e-government survey, the use of social media by governments tripled from 2010 to 2012, and rose by another 50% in 2014 alone [1]. Because the public health sector represents a major area of government expenditure for most countries, and while the general literature on social media for e-government may contain transferrable insights, it is important to examine how the use of social media for e-government has been approached in this particular context.

### Defining and Classifying e-Government

Although e-government (or eGovernment) is often used as a generic label for digitally mediated government, there is some variation in the use of this term. For example, it has been described to as “analogous to e-commerce, which allows businesses to transact with each other more efficiently and brings customers closer to businesses” [2] and as a means to transform governments’ relationships with citizens (government-to-citizen and/or citizen-to-government), employees (government-to-employees and/or employees-to-government), nonprofit organizations (government-to-nonprofit and/or nonprofit-to-government), businesses (government-to-business and/or nonprofit-to-business), and other arms of government (government-to-government) [2,5,6]. The information flows depicted in parentheses above are derived from a taxonomy originally developed by Fang [7].

In 2010, Linders et al (as cited in [8]) suggested several dimensions related to the objectives and intended outcomes underlying the use of social media and how these might affect the work of governmental agencies. These are as follows:

- “Democratic participation and engagement, through which social media technologies are used to involve the public in government decision processes, to foster participatory dialog and policy development and implementation.
- Co-production, through which governments and the public jointly develop, design, and deliver government services to improve service quality, delivery, and responsiveness.
- Crowdsourcing solutions and innovations, whereby governments seek public knowledge and talent to develop innovative solutions to large-scale societal issues.
- Transparency and accountability, through which government is open and transparent regarding its operations to build trust and foster accountability” [8].

Although variants of these taxonomies exist (eg, Linders [9] breaks down his co-production theme into “citizen sourcing,” “government as a platform,” and “do-it-yourself government”), these two broad frameworks [8,9] are useful for interpreting the results of our review and will be taken into account during this process.

Concepts such as “open government” and “e-governance” also overlap with e-government in important ways, such as through a shared objective for transparency [10]; however, each has somewhat unique connotations and communities of practice. For example, the former emphasizes online public access to government documents and statistics, whereas the latter emphasizes legal and regulatory requirements for effective digital services [11], including e-government [12].

Although there is a body of research describing the adoption of social media by public sector actors such as local authorities [13,14], central and federal governments [6,15], cities [16,17], and municipalities [18], many authors claim that little is known about how these technologies are used by public health organizations [19,20]. Moreover, to the best of our knowledge, no study has specifically investigated the adoption and use of social media by public health organizations, taking the perspective that they are also part of government [21].

### e-Government and the Public Health Sector

Determining the scope of the public health sector, in terms of its relationship with concepts of government and e-government, is also challenging. Within medicine, *public health* itself is regarded as a distinct discipline, involving the delivery of population-scale health interventions, such as prevention, screening, wellness, maternal and child health services, surveillance of diseases and risks, and scientific research aimed at understanding and improving the health of populations. By contrast, the *public health sector* is a much larger proposition, encompassing government departments of health, special government agencies tasked with public health activities, health care delivery organizations operating within the public sector, and the networks of voluntary and private-sector organizations that contribute to these activities, also collectively referred to as the *public health system* [22]. Although most countries have an underpinning government health system, the private sector plays a more dominant role in some than in others, due to historical, political, economic, and philosophical differences, which have affected the prioritization of market choice versus social equity [23]. For example, definitions of public health arising from the United Kingdom (operating largely in accordance with the Beveridge Social Insurance Model) emphasize the role of the government, whereas those from the United States (operating to a Mixed Market-Driven Model) tend to place a greater emphasis on the private sector; however, the World Health Organization’s definition encompasses all varieties of health systems, as illustrated by the examples shown in Textbox 1. From the perspective of our review topic, this complexity presents a challenge for bridging concepts of “public health sector” and “e-government,” and is likely to affect both the description of relevant studies in the literature and our ability to interpret them within an e-government framework. Nevertheless, governments typically maintain oversight and governance of medicine and health care in most countries, through their legal and regulatory powers, and it may therefore be legitimate to include nongovernmental organizations within the scope of our review. This will depend on the extent to which it is possible to adequately differentiate between governmental and nongovernmental public health activities in the included studies, which have not yet been subjected to detailed analysis or critical appraisal, and the choice will be defended in the main systematic review report.

**Sample definitions of public health/systems from the World Health Organization, the United States, and the United Kingdom.**

- World Health Organization: Global policy and surveillance, emphasis on lower income countries

“Public health refers to all organized measures (*whether public or private*) to prevent disease, promote health, and prolong life among the population as a whole. Its activities aim to provide conditions in which people can be healthy and focus on entire populations, not on individual patients or diseases. Thus, *public health is concerned with the total system* and not only the eradication of a particular disease” [24].

- United States: Mixed health care economy, largely private sector. Health care as service

Government (Centers for Disease Control and Prevention, Department of Health and Human Services): “Public health systems include *all public, private, and voluntary* entities that contribute to the delivery of essential public health services within a jurisdiction” [25].

Foundation established by government (CDC Foundation): “Public health is the *science* of protecting and improving the health of families and communities through promotion of healthy lifestyles, research for disease and injury prevention and detection and control of infectious diseases. Overall, public health is concerned with protecting the health of entire populations. These populations can be as small as *a local neighborhood, or as big as an entire country or region of the world*” [26].

- United Kingdom: Predominantly public sector, with optional private services. Health care as universal right

Government (Department of Health): “Public health is about helping people to stay healthy, and protecting them from threats to their health. The government wants everyone to be able to make healthier choices, regardless of their circumstances, and to minimise the risk and impact of illness” [27].

National Professional Society (The UK’s Faculty of Public Health): “Public Health is the science and art of promoting and protecting health and well-being, preventing ill-health and prolonging life through the organised efforts of society....Public health is population based, emphasises collective responsibility for health—its protection and disease prevention—*recognises the key role of the state,* linked to a concern for the underlying socio-economic and wider determinants of health, as well as disease, and emphasises partnerships with all those who contribute to the health of the population” [28].

### Social Media and Health

The literature on social media in health is large and growing: for example, in 2011, the number of social media-related abstracts in PubMed for the years 2002-2011 was 1471, whereas in 2012, there were already 2,330 returns, according to an analysis by Gholami-Kordkheili et al in 2013 [29], and is likely to be considerably higher today. Industry analysts are regularly charting the uses of social media for health-related purposes, indicating growth trends and their impacts on the health care business sector [30], while academic and policy conferences devoted to understanding the social Web and social media in health are proliferating (eg, [31,32]). Although some studies have clearly described the uses of social media for delivering public health services (eg, [33,34]), or for enabling e-government (eg, [35]), the conceptual links between public health, e-government, and social media have not been well described in the literature.

### Formative Scoping

#### Analysis of Existing Systematic Reviews on Social Media in Health

To gain a better understanding of the relevant concepts, and to establish the need for a new review, we first sought to identify and analyze existing systematic reviews on social media in health. The high-level terms “Social Media,” “Systematic Review,” and “Health*” were used to search the MEDLINE electronic database. Generated returns (n=27) were then filtered to remove duplicates (n=5), articles found not to be systematic reviews (n=1), and nonrelevant publications (n=1). The relevant systematic or quasi-systematic reviews revealed by this search (n=20) and additional systematic reviews identified through snowballing from the reference lists of these articles or found among the returns generated by our systematic review search query described in [36] (n=16) are presented in Multimedia Appendix 1 (n=36). In each case, the review is summarized in terms of its data sources, context, and focus. In most cases, abstracts were used for this analysis; however, full-text review was also necessary in a few cases where abstracts did not contain sufficient detail to allow a judgment to be made.

The scope of the existing systematic reviews (see Multimedia Appendix 1) varies quite widely. Some aim to synthesize the results of existing interventional studies involving social media, some describe the literature on the uses of social media for purposes such as medical education or professional networking, and others examine the potential of social media for facilitating research through recruitment or secondary analysis of data. However, as can be seen from Multimedia Appendix 1, none is a comprehensive overview aimed at understanding specifically how social media are adopted and used by health organizations either in the public sector or more widely.

#### Comparing Social Media in e-Government Generally, and Within the Public Health System

We also wished to compare the concepts used to discuss social media in the e-government literature as a whole, and in the subset of e-government literature focused on health systems, because these represent somewhat different communities of practice. To do this, we analyzed samples of titles and abstracts from each corpus of literature.

For the former, the terms “e-government” OR “eGovernment” OR “government” AND “social media” OR “Facebook” OR “Twitter” OR “YouTube” were applied to the Scopus database, and the titles and abstracts of the 50 most highly cited articles were examined (hereinafter referred to as “generic e-government research”). For the latter, we examined the draft list of about 90 relevant abstracts and titles identified using our search protocol registered in PROSPERO [36] (hereinafter referred to as “health e-government research”).

**Figure 1:**
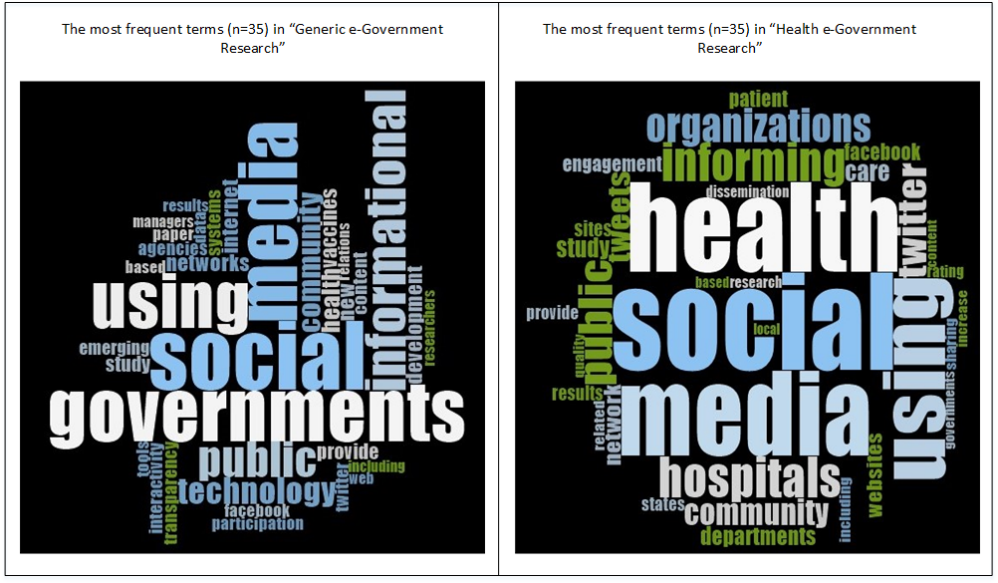
Words appearing in generic vs. health e-government research.

The “Word Frequency” query in the NVivo qualitative analysis software package was used to extract the 35 terms appearing most often within each corpus of titles and abstracts. These are depicted in Figure 1 as “word clouds” representing clusters of related terms, their frequency in the text and their proximity to one another. More than half of the most frequently used words are identical in both bodies of literature, suggesting a considerable degree of overlap, although “government” is a more dominant theme in the former and “health” in the latter.

**Figure 2:**
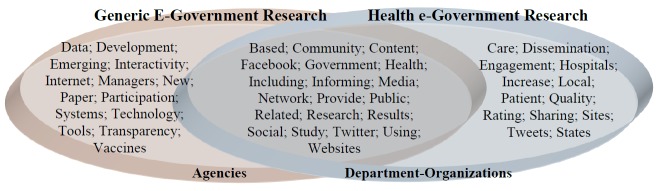
Analysis of terms used in “generic e-government” and “health e-government” research.

To gain a more nuanced understanding of the differences and overlaps between the generic and health-related e-governance literatures, we also manually plotted the individual terms according to the corpus of research in which they appeared, as shown in Figure 2.

The results summarized in Figure 2 indicate that, although there is overlap between the two corpuses of research, there are also some noteworthy differences. For example, governmental bodies are commonly referred to as “agencies” in the generic e-government literature and as “departments” and/or “organizations” in the health-related e-government literature. Within the generic e-government literature, concepts such as interaction, transparency, and public participation are prioritized, whereas in the health-related e-government literature, there is a greater emphasis on information sharing and dissemination, as well as engaging with the public to inform care quality improvement and obtain ratings of health services. Words like “using” appear frequently in both corpuses, as do other terms describing actions or high-level objectives such as “engagement,” as shown in Figures 1 and 2. This suggests that most of the existing research examining social media for e-government in general, and in the context of health systems, focuses on its use, rather than on its development, implementation processes, or impacts. This may reflect the tendency of government organizations to use off-the-shelf social media tools, reducing the requirement for design, although it may also indicate that organizations are engaging with these technologies in the optimistic belief that they will inevitably deliver benefits, rather than seeking to test this proposition. During our systematic review we will seek to identify formative and evaluative studies of social media as a service in the context of public health e-government.

Another interesting observation was that the explicit terms “e-government or eGovernment” did not appear in the corpus of abstracts on social media in health, when we tried to search for it via NVivo’s “Text Search” query, despite the high frequency of the single term “government,” although it was explicit in the generic e-government literature. In the next phase of our research, we will analyze the full text of included articles derived from each corpus, to examine whether this finding still holds, as well as to establish the overlaps with the related concepts of “open government” and “e-governance,” which we had expected to see independently among the most frequently used terms, at least within the corpus of generic e-government research (although the related term “transparency” appeared).

### Systematic Review Objectives

- To capture the corpus of published studies on the uses of social media by public health agencies or services, at the regional or national levels, in different countries;
- To classify the objectives for which social media have been deployed in these contexts, and the methods used to achieve them, as explicitly stated by the authors or deduced from the published descriptions;
- To analyze and synthesize evidence of the uptake and use of social media by various public sector health organizations and agencies worldwide and their impacts on a range of outcomes.

## Methods

### Data Sources and Search Methods

MEDLINE, Cochrane Library, Web of Science, and Scopus international electronic databases were searched on July 12, 2015, using the following search terms/query: (“e-government” OR “government” OR “department” OR “organization” OR “agenc*” OR “hospital*” OR “clinic*”) AND (“social media” OR “Facebook” OR “Twitter” OR “YouTube”) AND (“health” OR “Healthcare”). These databases were chosen for this systematic review because they cover a wide range of disciplines, including public health, medicine, technology, business, and social sciences. The search was not restricted to language, but the article selection was restricted to publication date; only studies published between January 1, 2004, and July 12, 2015, were included in this review. The year 2004 has been chosen as a starting point, since this was when Facebook, the most widely used social media website, was created. Relevant gray literature sources such as World Health Organization reports and working papers (searched via World Health Organization’s Institutional Repository for Information Sharing), and industry (eg, consultancy and marketing) reports (searched via Google search engine) were also searched to identify relevant publications.

### Studies Screening and Selection

**Figure 3:**
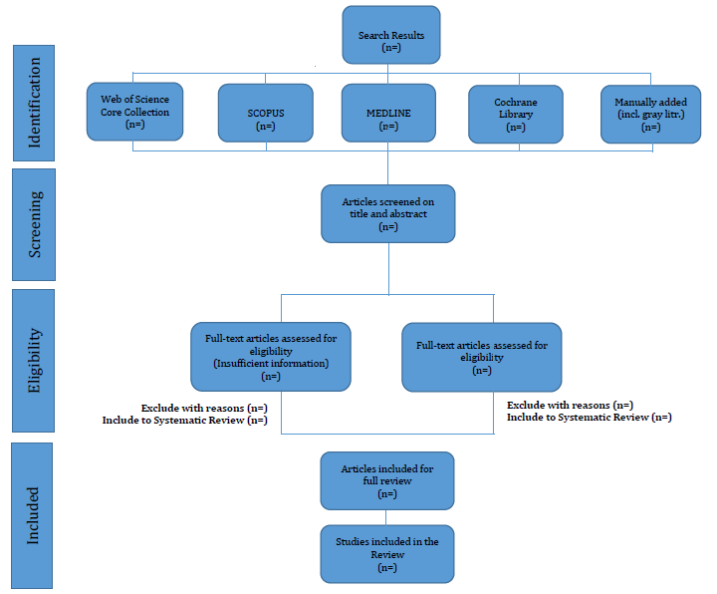
PRISMA flow diagram illustrating literature search and articles selection process.

One reviewer (AT) has independently screened generated citations with the help of EPPI-Reviewer 4 systematic review software (EPPI-Reviewer), and will extract the data from the qualified articles. Another author (CP) will then check this work to ensure accuracy. In case of any disagreement between reviewers during this stage, the arbitration of the third reviewer (MF) will be sought to resolve it. A PRISMA [37] flow diagram (Figure 3) will be created to track the study selection process and ensure transparency and replicability of the review. The final PRISMA diagram will specify the missing “n=” values, which will be the outcome of our analysis.

### Eligible Participants

Public sector health organizations that have deployed social media as a part of their e-government strategy including, but not limited health agencies, governmental departments and ministries. Public (state-funded) hospitals (when defined as such by publications’ authors) will be also included in this review, to see whether there are any similarities or differences in how they use social media compared with other public health organizations.

### Eligible Study Designs

This is not a review of clinical trials and our inclusion criteria encompass all types of study designs. However, based on our preliminary scoping work, we expect that most studies will be exploratory in nature. The inclusion and exclusion criteria are presented in Textbox 2.

**Inclusion and exclusion criteria.**

Inclusion criteria

- Academic or industry research with a primary focus on the adoption and use of social media by public sector health organizations at the regional or national levels. For example, studies that focused on social media adoption by government departments of public health, regional health authorities, state-funded (public) hospitals or other government-sponsored agencies with a public health remit.
- Studies focused on the most popular social media sites, such as Facebook, Twitter, and YouTube [38-40].
- Studies published between January 1, 2004, and July 12, 2015.

Exclusion criteria

- Studies focused on private sector health organizations.
- Studies focused on individual units within public sector health organizations, such as emergency care or cardiology services and individual clinics.
- Studies primarily focused on social media for health surveillance or research.
- Studies focused on uses by specific professional or patient groups (eg, diabetes specialists or patients) or by individuals.
- Studies published before January 1, 2004, and after July 12, 2015.

### Outcome Measures

#### Primary Outcomes

Indicators of uptake and use of social media and reported impacts on organizational transparency, efficiency, or effectiveness.

#### Secondary Outcomes

Perceived increase of government-to-government, government-to-citizen, government-to-business, and government-to-employee interaction, engagement, and satisfaction.

### Data Analysis and Synthesis

We plan to narratively summarize and synthesize review results, taking into consideration the likelihood of heterogeneity of organizations and social media types studied, as well the designs of those studies. The following information is planned to be extracted from the studies that meet the inclusion criteria:

- authors/year;
- setting: country; organization; size; year;
- social media used;
- stated objective/purpose for using social media;
- research question;
- theoretical basis;
- study design and scope;
- outcomes examined, if relevant;
- main findings;
- conclusion/comments.

### Critical Appraisal Techniques

Critical Appraisal Skills Programme checklist [41] will be used to assess the quality of the included studies.

## Results

A comprehensive search strategy was created, tested, and applied to the aforementioned databases. Generated citations have been uploaded to EPPI-Reviewer, where they are being screened and coded. The work on this review is planned to be completed in the beginning of 2016 and its results will be presented at international academic conferences and published in a respected peer-reviewed journal.

## Discussion

Social media present potentially valuable opportunities for public sector health organizations to meet the objectives of e-government, and social media are increasingly being used by such organizations. However, little is known about how this is being achieved in practice and no previous systematic reviews have sought to synthesize the relevant evidence. Undertaking a systematic review in this area is complicated by variability in the terms used to describe the concepts of e-government and public health, although taxonomies are available to support interpretation of the literature. The priorities for using social media in the health sector appear to be somewhat different from those of the generic e-government agenda, although the terms and concepts referred to in the relevant research literature overlap significantly. The preliminary observation that most research articles within the scope of our systematic review focus on usage alone suggests that public health organizations may be taking it for granted that social media will deliver benefits, rather than attempting to track their objectives for adoption or evaluate their impacts. A more detailed analysis of articles meeting the inclusion criteria will help to improve our understanding of this literature and inform the development of recommendations for research and practice. Undertaking this scoping review has provided valuable insights to guide the design and interpretation of the research literature with reference to a wider range of conceptual, disciplinary, and international considerations, supporting previous recommendations for the planning of systematic reviews [42-44] as well as testing innovative methods of mapping relevant terms and concepts.

## Appendix

Multimedia Appendix 1 Existing systematic reviews on social media in health care.
