# Supplementary material for: Social Media for e-Government in the Public Health Sector: Protocol for a Systematic Review
Source: JMIR Res Protoc. 2016 Mar 11;5(1):e42. doi: 10.2196/resprot.5421 (PMC4808239; doi:10.2196/resprot.5421)
Supplement: Multimedia Appendix 1 [file resprot_v5i1e42_app1.pdf]

MULTIMEDIA APPENDIX 1. Existing systematic reviews on social media in health care

N	Systematic Review	Review Aim/Objective/Main Focus	Number of Databases used
1	Cotter AP, Durant N, Agne AA, Cherrington AL. Internet interventions to support lifestyle modification for diabetes management: a systematic review of the evidence. J Diabetes Complications. 2014;28(2):243–51.	To identify studies that used Internet based interventions to promote lifestyle modification among adults with type 2 diabetes.	1
2	Sampson M, Cumber J, Li C, Pound CM, Fuller A, Harrison D. A systematic review of methods for studying consumer health YouTube videos, with implications for systematic reviews. PeerJ. 2013;12;1:e147.	To examine the methods used in reviews of YouTube consumer health videos to identify trends and best practices.	1
3	Gholami-Kordkheili F, Wild V, Strech D. The impact of social media on medical professionalism: a systematic qualitative review of challenges and opportunities. J Med Internet Res. 2013;15(8):e184.	To analyze the full spectrum of (1) social media-related challenges imposed on medical professionalism and (2) social media-related opportunities to both undermine and improve medical professionalism.	1
4	Yonker LM, Zan S, Scirica CV, Jethwani K, Kinane TB. “Friending” teens: systematic review of social media in adolescent and young adult health care. J Med Internet Res. 2015;17(1):e4.	To identify research on the use of social media for interacting with adolescents and young adults in order to achieve positive health outcomes.	1
5	Madathil KC, Rivera-Rodriguez AJ, Greenstein JS, Gramopadhye AK. Healthcare information on YouTube: A systematic review. Health Informatics J. 2015;21(3):173–94.	To review the peer-reviewed literature addressing the healthcare information available on YouTube.	2
6	Capurro D, Cole K, Echavarría MI, Joe J, Neogi T, Turner AM. The use of social networking sites for public health practice and research: a systematic review. J Med Internet Res. 2014;16(3):e79.	To identify the use of social networking sites (SNSs) for public health research and practice and to identify existing knowledge gaps.	3
7	Velasco E, Agheneza T, Denecke K, Kirchner G, Eckmanns T. Social media and internet-based data in global systems for public health surveillance: a systematic review. Milbank Q. 2014;92(1):7–33.	To explore how useful are data from social media and the Internet, and what is the potential to enhance surveillance?	3
8	Alshaikh F, Ramzan F, Rawaf S, Majeed A. Social network sites as a mode to collect health data: a systematic review. J Med Internet Res. 2014;16(7):e171.	To systematically review the available literature and explore the use of SNS as a mode of collecting data for health research.	3
9	Koskan A, Klasko L, Davis SN, Gwede CK, Wells KJ, Kumar A, et al. Use and taxonomy of social media in cancer-related research: a systematic review. Am J Public Health. 2014;104(7):e20–37.	To examine the use and taxonomy of social media in cancer-related studies.	4
10	Tuong W, Larsen ER, Armstrong AW. Videos to influence: a systematic review of effectiveness of video-based education in modifying health behaviors. J Behav Med. 2014;37(2):218–33.	To examine the effectiveness of videos in modifying health behaviors.	4
11	Montague E, Perchonok J. Health and wellness technology use by historically underserved health consumers: systematic review. J Med Internet Res. 2012;14(3):e78.	To examine ways in which technology is being used by historically underserved populations to decrease health disparities through facilitating or improving health care access and health and wellness outcomes.	4
12	El-Gayar O, Timsina P, Nawar N, Eid W. Mobile applications for diabetes self-management: status and potential. J Diabetes Sci Technol. 2013;7(1):247–62.	To determine, in a systematic review, whether diabetes applications have been helping patients with type 1 or type 2 diabetes self-manage their condition and to identify issues necessary for large-scale adoption of such interventions.	4

13	Roberts MJ, Perera M, Lawrentschuk N, Romanic D, Papa N, Bolton D. Globalization of Continuing Professional Development by Journal Clubs via Microblogging: A Systematic Review. J Med Internet Res. 2015;17(4):e103.	To evaluate the current state of social media–facilitated journal clubs, specifically Twitter, as an example of continuing professional development.	5
14	Badr H, Carmack CL, Diefenbach MA. Psychosocial interventions for patients and caregivers in the age of new communication technologies: opportunities and challenges in cancer care. J Health Commun. 2015;20(3):328–42.	To explore the extent that Interactive Health Communication Technologies have been used in dyadic interventions in cancer and to explore whether they facilitate patient-caregiver communication and outcomes.	5
15	Laranjo L, Arguel A, Neves AL, Gallagher AM, Kaplan R, Mortimer N, et al. The influence of social networking sites on health behavior change: a systematic review and meta-analysis. J Am Med Inform Assoc. 2015;22(1):243–56.	To evaluate the use and effectiveness of interventions using SNSs to change health behaviors.	5
16	Stellefson M, Chaney B, Barry AE, Chavarria E, Tennant B, Walsh-Childers K, et al. Web 2.0 chronic disease self-management for older adults: a systematic review. J Med Internet Res. 2013;15(2):e35.	To review the planning, implementation, and overall effectiveness of Web 2.0 self-management interventions for older adults (mean age ≥ 50) with one or more chronic disease(s).	6
17	Highton-Williamson E, Priebe S, Giacco D. Online social networking in people with psychosis: A systematic review. Int J Soc Psychiatry. 2015;61(1):92–101.	To explore available evidence on the use of online social networking in people with psychosis.	6
18	Newbold BK, Campos S. Media and Social Media in Public Health Messages: A Systematic Review. McMaster University of Environment and Health. 2011. http://www.mcmaster.ca/mihe/documents/publications/Social%20Media%20Report.pdf . Accessed 8 Nov 2015.	To determine the efficacy of traditional and social media in delivering public health messages.	6
19	Park BK, Calamaro C. A systematic review of social networking sites: innovative platforms for health research targeting adolescents and young adults. J Nurs Scholarsh. 2013;45(3):256–64.	To review the evidence to determine if SNS are effective tools for health research in the adolescent and young adult populations.	7
20	Schein R, Wilson K, Keelen J. Literature review on effectiveness of the use of social media: A report for Peel Public Health. 2010. https://www.peelregion.ca/health/resources/pdf/socialmedia.pdf. Accessed 8 Nov 2015.	This report targets original research, case studies, reviews, and commentaries related to public health communication.	7
21	Guse K, Levine D, Martins S, Lira A, Gaarde J, Westmorland W, et al. Interventions using new digital media to improve adolescent sexual health: a systematic review. J Adolesc Health. 2012;51(6):535–43.	To summarize the currently published evidence base on the effectiveness of new digital media-based sexual health interventions for adolescents aged 13–24 years.	8
22	Maher CA, Lewis LK, Ferrar K, Marshall S, De Bourdeaudhuij I, Vandelanotte C. Are health behavior change interventions that use online social networks effective? A systematic review. J Med Internet Res. 2014;16(2):e40.	To systematically review the current level of evidence regarding the effectiveness of online social network health behavior interventions.	8
23	Cartledge P, Miller M, Phillips B. The use of social-networking sites in medical education. Med Teach. 2013;35(10):847–57.	To ascertain if "SNSs have been used successfully in medical education to deliver educational material", and whether "healthcare professionals, and students, are engaging with SNSs for educational purposes".	8
24	Nef T, Ganea RL, Müri RM, Mosimann UP. Social networking sites and older users - a systematic review. Int Psychogeriatr. 2013;25(7):1041–53.	To give an overview of existing scientific literature on social networking in older users.	8
25	Amon KL, Campbell AJ, Hawke C, Steinbeck K. Facebook as a recruitment tool for adolescent health research: a systematic review. Acad Pediatr. 2014;14(5):439–47.e4.	To conduct a systematic review of the literature on the use of Facebook to recruit adolescents for health research.	9

26	Batt-Rawden S, Flickinger T, Weiner J, Cheston C, Chisolm M. The role of social media in clinical excellence. Clin Teach. 2014;11(4):264–9.	To identify examples of how social media may be used to help promote the achievement of clinical excellence in medical learners.	9
27	Alvarez-Jimenez M, Alcazar-Corcoles MA, González-Blanch C, Bendall S, McGorry PD, Gleeson JF. Online, social media and mobile technologies for psychosis treatment: a systematic review on novel user-led interventions. Schizophr Res. 2014;156(1):96–106.	To systematically compile and analyze the evidence on the acceptability, feasibility, safety and benefits of online and mobile-based interventions for psychosis.	9
28	Moorhead SA, Hazlett DE, Harrison L, Carroll JK, Irwin A, Hoving C. A new dimension of health care: systematic review of the uses, benefits, and limitations of social media for health communication. J Med Internet Res. 2013;15(4):e85.	To identify the uses, benefits, and limitations of social media for health communication among the general public, patients, and health professionals, and identify current gaps in the literature to provide recommendations for future health communication research.	10
29	Hamm MP, Shulhan J, Williams G, Milne A, Scott SD, Hartling L. A systematic review of the use and effectiveness of social media in child health. BMC Pediatr. 2014;14:138.	To determine: 1) for what purposes social media is being used in child health and its effectiveness; and 2) the attributes of social media tools that may explain how they are or are not effective.	11
30	Williams G, Hamm MP, Shulhan J, Vandermeer B, Hartling L. Social media interventions for diet and exercise behaviours: a systematic review and meta-analysis of randomized controlled trials. BMJ Open. 2014;4:e003926.	To conduct a systematic review of randomized controlled trials (RCTs) examining the use of social media to promote healthy diet and exercise in the general population.	11
31	Simmons C, Rajmohan Y, Poonja Z, Adilman R. Social media in cancer care: opportunities to improve care in locally advanced breast cancer. Curr Opin Support Palliat Care. 2014;8(1):77–82.	To examine the current data supporting use of social media in breast cancer clinical care.	Not specified*
32	Rahim FA, Ismail Z, Samy GN. Information Privacy Concerns in the Use of Social Media Among Healthcare Practitioners: A Systematic Literature Review. Advanced Science Letters. 2014;20(10-12):2176–9.	To design a proposed information privacy concerns model in the use of social media among healthcare practitioners.	Not specified*
33	Jones K, Eathington P, Baldwin K, Sipsma H. The impact of health education transmitted via social media or text messaging on adolescent and young adult risky sexual behavior: a systematic review of the literature. Sex Transm Dis. 2014;41(7):413–9.	To examine the effectiveness of social media and text messaging interventions designed to increase sexually transmitted disease (STD) knowledge, increase screening/testing, decrease risky sexual behaviors, and reduce the incidence of STDs among young adults aged 15 through 24 years.	Not specified*
34	MacLeod MG, Hoppe DJ, Simunovic N, Bhandari M, Philippon MJ, Ayeni OR. YouTube as an information source for femoroacetabular impingement: a systematic review of video content. Arthroscopy. 2015;31(1):136–42.	To assess the quality of information available on YouTube regarding femoroacetabular impingement.	YouTube
35	Wittenberg-Lyles E, Parker Oliver D, Demiris G, Swarz J, Rendo M. YouTube as a tool for pain management with informal caregivers of cancer patients: a systematic review. J Pain Symptom Manage. 2014;48(6):1200–10.	To explore the availability and characteristics of instructional cancer pain management videos on YouTube and determine to what extent these videos addressed the role of informal caregivers in cancer pain management.	YouTube
36	Harrison D, Sampson M, Reszel J, Abdulla K, Barrowman N, Cumber J, et al. Too many crying babies: a systematic review of pain management practices during immunizations on YouTube. BMC Pediatr. 2014;14:134.	To review the content of YouTube videos showing infants being immunized to ascertain parents' and health care professionals' use of pain management strategies, as well as to assess infants' pain and distress.	YouTube

*Databases used were not specified in the Abstract and publication full text was not found.
